# Ethanol Modulates Glutamatergic Transmission and NMDAR-Mediated Synaptic Plasticity in the Agranular Insular Cortex

**DOI:** 10.3389/fphar.2018.01458

**Published:** 2018-12-18

**Authors:** Joel E. Shillinglaw, Richard A. Morrisett, Regina A. Mangieri

**Affiliations:** Division of Pharmacology and Toxicology, College of Pharmacy, The University of Texas at Austin, Austin, TX, United States

**Keywords:** electrophysiology, ethanol, glutamate, synaptic plasticity, insular cortex, mouse

## Abstract

The agranular insular cortex (AIC) has recently been investigated by the alcohol field because of its connectivity to and modulatory control over limbic and brainstem regions implicated in alcohol use disorder (AUD), and because it has shown involvement in animal models of alcohol drinking. Despite evidence of AIC involvement in AUD, there has not yet been an examination of whether ethanol modulates glutamatergic and γ-amino-butyric acid (GABA)ergic synaptic transmission and plasticity in the AIC. Characterizing how the synaptic transmission and plasticity states of AIC cortical processing neurons are modulated by acute ethanol will likely reveal the molecular targets by which chronic ethanol alters AIC function as alcohol drinking transitions from controlled to problematic. Therefore, we collected brain slices from ethanol-naïve adult male mice, obtained whole-cell recording configuration in layer 2/3 AIC pyramidal neurons, and bath-applied ethanol at pharmacologically relevant concentrations during electrophysiological assays of glutamatergic and GABAergic synaptic transmission and plasticity. We found that ethanol inhibited electrically evoked N-methyl-D-aspartate receptor (NMDAR)-mediated excitatory post-synaptic currents (EPSCs) in a concentration-related fashion, and had little effect on evoked α-amino-3-hydrox-5-methylisoxazole-4-propionic acid-type receptor (AMPAR)-mediated EPSCs. Ethanol had no effect on spontaneous excitatory post-synaptic currents (sEPSCs) or inhibitory GABA_A_R-mediated post-synaptic currents (sIPSCs). We found that synaptic conditioning (low-frequency stimulation for 15 min at 1 Hz) induced a form of long-term depression (LTD) of evoked AMPAR-mediated EPSCs. The ability to induce LTD was inhibited by a non-selective NMDAR antagonist (DL-2-amino-5-phosphonovaleric acid), and also by acute, intoxicating concentrations of ethanol. Taken together these data suggest that the glutamate, but not GABA system in the AIC is uniquely sensitive to ethanol, and that in particular NMDAR-mediated processes in the AIC may be disrupted by pharmacologically relevant concentrations of ethanol.

## Introduction

Despite several years of preclinical research investigating the mechanisms underlying the transition from controlled to problematic alcohol drinking in order to develop future therapeutic approaches, alcohol use disorder (AUD) remains one of the most prevalent and costly health problems in the United States (Stahre et al., 2014; Sacks et al., 2015). Current pharmacological and behavioral treatments have achieved only moderate success largely due to their inability to decrease the vulnerability to relapse in abstinent addicts (Scofield et al., 2016). Therefore, investigating the neural networks implicated in the craving and relapse components of AUD is vital to developing more efficacious treatment options. Recent research suggests that deficits in interoceptive processing, or the processing and integration of physiological bodily states, may in part contribute to craving and relapse components of substance use disorders (Paulus and Stewart, 2014).

The agranular insular cortex (AIC) is a brain region implicated in interoceptive processing, and altered AIC function and output to subcortical limbic regions has been shown to mediate alcohol intake in animal models of AUD (Seif et al., 2013; Jaramillo et al., 2016, 2018a,b). Yet despite evidence for altered AIC function and output in AUD, there has been no investigation of whether the AIC is an ethanol-sensitive brain region in which basic synaptic functions are modulated by pharmacologically relevant concentrations of acute ethanol. It is widely accepted that the synapse is sensitive to ethanol, and that ethanol's major pharmacodynamic effects occur at least in part via its modulatory actions on the major fast excitatory and inhibitory neurotransmitter systems, glutamate and gamma-aminobutyric acid (GABA), respectively. However, these synaptic actions have been shown to be both brain region and concentration-dependent (Lovinger et al., 1990; Nie et al., 1994; Roberto et al., 2003; Kash et al., 2008; Weitlauf and Woodward, 2008; Badanich et al., 2013).

Moreover, the synaptic targets of acute ethanol have generally displayed opposing or compensatory effects after chronic ethanol exposure in animal models designed to mimic long-term alcohol abuse (Lovinger and Roberto, 2013). These compensatory effects of chronic ethanol on synaptic receptors have been shown to encode long-term alterations in glutamatergic and GABAergic transmission and to underlie, in part, aspects of AUD, such as withdrawal, tolerance and dependence (Jeanes et al., 2011; Lovinger and Roberto, 2013; Lovinger and Kash, 2015; Renteria et al., 2017). We therefore suggest that any synaptic target in the AIC that is sensitive to acute ethanol may be a target by which chronic ethanol disrupts AIC function as chronic ethanol shifts alcohol drinking from controlled to problematic.

For these reasons we investigated the effects of acute ethanol on pharmacologically isolated glutamatergic and GABAergic synaptic transmission, and an N-methyl D-aspartate-type glutamate receptor (NMDAR)-dependent glutamatergic synaptic plasticity in mouse AIC. We decided to investigate layer 2/3 pyramidal neurons since layer 2/3 of the cortex is generally considered the intracortical processing layer. We found that NMDAR-mediated currents were inhibited by pharmacologically relevant concentrations of ethanol. Conversely, alpha-amino-3-hydroxy-5-methylisoxazole-4-propionic acid-type glutamate receptor (AMPAR)-mediated currents were insensitive to ethanol. Ethanol had no effect on spontaneous excitatory post-synaptic currents (sEPSCs) or spontaneous inhibitory γ-amino-butyric acid receptor (GABAR)-mediated post-synaptic currents (sIPSCs). Our investigated form of synaptic plasticity, NMDAR-dependent long-term depression (LTD), was sensitive to pharmacologically relevant concentrations of ethanol. These findings are the initial demonstration that the AIC is a direct synaptic target for the actions of ethanol, and that glutamatergic transmission and plasticity, but not GABAergic transmission, is sensitive to pharmacologically relevant concentrations of acute ethanol.

## Methods

### Preparation of Brain Slices

Mice were briefly anesthetized with isoflurane, euthanized by decapitation, and then brains were rapidly extracted and placed in ice-cold oxygenated artificial cerebrospinal fluid (ACSF) containing the following (in mM): 210 Sucrose, 26.2 NaHCO_3_, 1 NaH_2_PO_4_, 2.5 KCl, 11 dextrose, bubbled with 95% O_2_/5% CO_2_. Coronal slices (230 to 250 μm thick) containing the most anterior portion of the AIC (anterior-posterior = +2.46 to +1.54) were then collected in ice-cold oxygenated ACSF using a Leica VT1000S vibrating microtome (Leica Corp., Bannockburn, IL). Slices were then transferred into an incubation solution containing the following (in mM): 120 NaCl, 25 NaHCO_3_, 1.23 NaH_2_PO_4_, 3.3 KCl, 2.4 MgCl_2_, 1.8 CaCl_2_, 10 dextrose, continuously bubbled with 95% O_2_/5% CO_2_; 32°C, and maintained in this solution at least 45 min prior to recording.

### Patch-Clamp Electrophysiology

Whole cell voltage clamp recordings were made in layer 2/3 pyramidal AIC neurons from anterior-posterior = +2.46 to +1.54. Pyramidal neurons were identified based on morphology (large, pyramidal shape) using a MRK200 Modular Imaging system (Siskiyou Corporation, Grants Pass, OR) mounted on a vibration isolation table. Passive electrical membrane properties for each cell at the beginning and end of each experiment are provided in Tables 1–7. Recordings were made in ACSF containing (in mM): 120 NaCl, 25 NaHCO_3_, 1.23 NaH_2_PO_4_, 3.3 KCl, 1.2 MgSO_4_, 2.0 CaCl_2_, and 10 dextrose unless otherwise noted, bubbled with 95% O_2_/5% CO_2_; 32°C, controlled by an in-line bath heather (Warner Instruments, Hamden, CT). The bath ACSF perfused brain slices at a rate of 2.0 mL/min. Recording electrodes (thin-wall glass, WPI Instruments, Sarasota FL) were made using a P-97 Flaming/Brown model micropipette puller (Sutter Instruments, San Rafael, CA) which produced electrodes of resistances from 3 to 6 MΩ. Series resistance (Rs) was monitored throughout the duration of each experiment and cells with Rs of over 30 MΩ or that changed over 20% over the course of the experiment were excluded from the analysis.

**Table 1:** Membrane properties for experiment shown in Figure 2.

	**Control**						**20 mM**						**40 mM**						**60 mM**						**80 mM**					
	**Start**			**End**			**Start**			**End**			**Start**			**End**			**Start**			**End**			**Start**			**End**		
**Cell**	**Cm (pF)**	**Rm (MΩ)**	**Ra (MΩ)**	**Cm (pF)**	**Rm (MΩ)**	**Ra (MΩ)**	**Cm (pF)**	**Rm (MΩ)**	**Ra (MΩ)**	**Cm (pF)**	**Rm (MΩ)**	**Ra (MΩ)**	**Cm (pF)**	**Rm (MΩ)**	**Ra (MΩ)**	**Cm (pF)**	**Rm (MΩ)**	**Ra (MΩ)**	**Cm (pF)**	**Rm (MΩ)**	**Ra (MΩ)**	**Cm (pF)**	**Rm (MΩ)**	**Ra (MΩ)**	**Cm (pF)**	**Rm (MΩ)**	**Ra (MΩ)**	**Cm (pF)**	**Rm (MΩ)**	**Ra (MΩ)**
1	90	155	16	89	184	16	291	76	12	281	51	14	241	179	21	237	176	21	224	143	15	194	125	18	144	121	25.1	151	173	23
2	247	181	19	246	185	20	239	12	14	217	139	15	266	82	21	232	92	22	117	160	29	116	170	29	182	116	19.2	187	133	19
3	190	351	21	183	332	24	220	224	13	208	64	16	272	146	14	240	125	14	210	93	14	219	43	12	223	213	14.1	222	213	14
4	182	149	11	186	123	13	159	213	21	146	202	23	262	218	10	251	174	12	224	120	15	210	115	16	172	158	19.7	158	144	22
5							211	117	16	206	110	17	278	134	16	277	142	15	199	252	12	194	266	12	123	256	17.1	116	96	18
6							139	104	27	140	114	26	222	101	25	213	48	23	152	114	24	165	156	21	203	200	16	203	197	16
7							64	239	13	65	279	13	212	82	25	207	64	28	73	101	14	70	109	14	180	174	18.5	160	139	21
8							56	172	18	57	195	18													74	397	12	71	550	11

**Table 2:** Membrane properties for experiment shown in Figure 3.

	**Control**						**40 mM**						**60 mM**						**80 mM**					
	**Start**			**End**			**Start**			**End**			**Start**			**End**			**Start**			**End**		
**Cell**	**Cm (pF)**	**Rm (M Ω)**	**Ra (M Ω)**	**Cm (pF)**	**R m (M Ω)**	**Ra (M Ω)**	**C m (pF)**	**Rm (M Ω)**	**Ra (M Ω)**	**Cm (pF)**	**Rm (M Ω)**	**Ra (M Ω)**	**Cm (p F)**	**Rm (M Ω)**	**Ra (M Ω)**	**Cm (p F)**	**Rm (M Ω)**	**Ra (M Ω)**	**C m (p F)**	**R m (M Ω)**	**Ra (M Ω)**	**Cm (pF)**	**Rm (M Ω)**	**Ra (M Ω)**
1	262	86	22	268	69	22	140	56	27	145	36	25	155	81	28	174	80	29	200	88	28	200	83	29
2	190	107	25	190	112	28	190	128	29	197	128	28	112	53	28	115	46	29	158	144	24	156	143	23
3	192	101	24	173	105	29	225	86	22	228	84	26	199	79	16	185	78	18	114	154	24	127	134	22
4	178	90	18	189	81	21	207	107	16	193	61	19	106	137	25	99	148	24	181	106	24	196	112	20
5							199	156	18	187	128	21	279	38	21	306	24	19	117	304	17	113	274	18
6							132	198	23	145	198	26							118	145	19	111	180	20
7																			224	68	25	189	65	26
8																			168	101	17	193	171	18
9																			173	142	20	149	230	18

**Table 3:** Membrane properties for experiment shown in Figure 4.

	**Control**						**50 mM**					
	**Start**			**End**			**Start**			**End**		
**Cell**	**Cm (pF)**	**Rm (MΩ)**	**Ra (MΩ)**	**Cm (pF)**	**Rm (MΩ)**	**Ra (MΩ)**	**Cm (pF)**	**Rm (MΩ)**	**Ra (MΩ)**	**Cm (pF)**	**Rm (MΩ)**	**Ra (MΩ)**
1	233	129	23	215	127	25	228	179	20	166	70	17
2	205	114	27	192	127	29	159	341	23	146	74	21
3	178	198	23	103	115	25	202	110	18	186	122	20
4	222	95	26	226	98	25	210	164	25	188	218	27
5	216	212	16	207	70	16	259	108	21	222	119	25
6	71	416	27	65	458	24	300	107	19	256	88	19
7	256	78	23	229	96	25	89	59	22	80	57	18
8	206	23	24	216	32	25	205	143	26	190	116	26
9							137	107	28	145	101	26

**Table 4:** Membrane properties for experiment shown in Figure 5.

	**Control**						**50 mM**					
	**Start**			**End**			**Start**			**End**		
**Cell**	**Cm (pF)**	**Rm (MΩ)**	**Ra (MΩ)**	**Cm (pF)**	**Rm (MΩ)**	**Ra (MΩ)**	**Cm (pF)**	**Rm (MΩ)**	**Ra (MΩ)**	**Cm (pF)**	**Rm (MΩ)**	**Ra (MΩ)**
1	224	200	20	192	176	22	242	98	23	223	106	25
2	216	116	14	138	146	14	63	224	13	96	180	15
3	169	107	24	131	124	23	160	112	28	193	138	26
4	198	106	20	171	98	23	237	68	20	230	101	20
5	79	122	29	94	196	28	231	81	26	209	104	27
6	203	78	19	199	61	20	206	91	14	175	109	15
7							240	81	24	260	94	20
8							148	130	22	136	128	21
9							134	174	25	141	265	23
10							130	171	28	120	178	29
11							175	174	15	139	146	18

**Table 5:** Membrane properties for experiment shown in Figure 6.

	**Naive**						**Naïve w/paired pulse**					
	**Start**			**End**			**Start**			**End**		
**Cell**	**Cm (pF)**	**Rm (MΩ)**	**Ra (MΩ)**	**Cm (pF)**	**Rm (MΩ)**	**Ra (MΩ)**	**Cm (pF)**	**Rm (MΩ)**	**Ra (MΩ)**	**Cm (pF)**	**Rm (MΩ)**	**Ra (MΩ)**
1	284	48	20	281	59	22	159	58	24	71	40	22
2	129	206	25	124	228	20	202	70	20	169	65	23
3	187	100	18	163	90	17	233	94	24	215	54	25
4	286	118	18	235	492	20	203	122	27	197	114	29
5	158	112	28	188	102	29	200	117	28	182	130	29
6	165	74	16	182	85	14	145	224	21	140	134	24
7	108	102	11	63	133	11						
8	209	76	28	162	81	25						
9	199	126	28	194	116	29						
10	192	170	27	210	145	26						
11	203	95	18	112	62	16						
12	171	139	20	160	89	23						

**Table 6:** Membrane properties for experiment shown in Figure 7.

	**DL-APV**					
	**Start**			**End**		
**Cell**	**Cm (pF)**	**Rm (MΩ)**	**Ra (MΩ)**	**Cm (pF)**	**Rm (MΩ)**	**Ra (MΩ)**
1	177	192	29	176	116	29
2	138	107	21	91	123	21
3	154	91	21	161	97	24
4	194	286	19	183	308	19
5	174	195	29	192	208	26
6	184	92	28	179	83	25
7	331	79	17	279	117	193

**Table 7:** Membrane properties for experiment shown in Figure 8.

	**20mM**						**40mM**						**60mM**					
	**Start**			**End**			**Start**			**End**			**Start**			**End**		
**Cell**	**Cm (pF)**	**Rm (MΩ)**	**Ra (MΩ)**	**Cm (pF)**	**Rm (MΩ)**	**Ra (MΩ)**	**Cm (pF)**	**Rm (MΩ)**	**Ra (MΩ)**	**Cm (pF)**	**Rm (MΩ)**	**Ra (MΩ)**	**Cm (pF)**	**Rm (MΩ)**	**Ra (MΩ)**	**Cm (pF)**	**Rm (MΩ)**	**Ra (MΩ)**
1	113	46	19	74	74	19	150	108	22	138	102	21	133	72	17	94	35	20
2	279	139	18	295	167	17	125	77	28	110	39	25	202	106	28	195	79	29
3	276	170	18	259	159	20	241	98	23	219	123	25	258	68	16	290	86	19
4	245	88	23	220	77	27	155	140	27	146	137	29	222	95	27	298	94	28
5	134	116	27	84	130	24	125	121	16	118	150	18	186	76	25	151	38	26
6	199	80	21	123	34	18	217	81	18	148	115	19	204	81	20	174	72	23
7	247	94	19	220	69	21							181	152	19	167	107	21

All chemicals, unless otherwise noted, were obtained from Sigma-Alrich or Tocris Bioscience with the exception of ethanol, which was obtained from Pharmco-Aaper. Multiple cells per brain slice were sometimes recorded from in glutamatergic and GABAergic transmission experiments, but only when the first cell recorded from was assigned to the time and sham solution exchange control condition. Thus, the final recording for each brain slice occurred once the slice was exposed to ethanol. For LTD experiments, only one cell per brain slice was used.

### Evoked Glutamatergic Transmission

For evoked excitatory (NMDAR-mediated) post-synaptic currents, recording electrodes were filled with (in mM): 120 CsMeSO_4_, 15 CsCl, 8 NaCl, 10 HEPES, 0.2 EGTA, 10 TEA-Cl, 4 Mg-ATP, 0.3 Na-GTP, 0.1 Spermine, and 5 QX-314-Cl. DNQX (20 μM) was added to the recording ACSF to block AMPA receptors, along with picrotoxin (50 μM) to block GABA_A_ receptors. The recording ACSF for evoked NMDAR-mediated experiments contained 1.0 mM MgSO_4_, and EPSCs were evoked by local stimulation while holding the post-synaptic membrane voltage at −40 mV for 2.4 s. For evoked excitatory (AMPAR-mediated) post-synaptic currents, recording electrodes were filled with (in mM): 120 K-gluconate, 10 KCl, 10 HEPES, 2 MgCl_2_, 1 EGTA, 2 Mg-ATP, and 0.3 Tris-GTP. DL-APV (100 μM) was added to the recording ACSF to block NMDA receptors, along with picrotoxin (50 μM). Neurons in evoked AMPAR-mediated experiments were held at −70 mV for the entirety of the experiment. For both evoked AMPAR-mediated and NMDAR-mediated post-synaptic current experiments, standard evoked EPSCs were established for at least 8 min (at 0.025 Hz) to ensure stable recordings, followed by 10 min periods of ethanol treatment and ethanol washout. Additional validation experiments were conducted to confirm that the currents under investigation were mediated by the receptors of interest. Evoked NMDA-mediated synaptic currents were reduced in amplitude by ≈78% by 100 μM DL-APV, and evoked AMPAR-mediated synaptic currents were reduced in amplitude by ≈93% by 20 μM DNQX (data not shown).

### Spontaneous Glutamatergic and GABAergic Transmission

For spontaneous excitatory post-synaptic currents (sEPSCs), recording electrodes were filled with (in mM): 135 KMeSO_4_, 12 NaCl, 0.5 EGTA, 10 HEPES, 2 Mg-ATP, and 0.3 Tris-GTP. Picrotoxin (50 μM) was added to the recording ACSF. For spontaneous inhibitory post-synaptic currents (sIPSCs), recording electrodes were filled with (in mM): 120 CsCl, 10 HEPES, 2 MgCl_2_, 1 EGTA, 2 Mg-ATP, 0.3 Tris-GTP, and 1 QX-314. Kynurenic acid (1 mM) was added to the recording ACSF to block AMPA and NMDA receptors. For both spontaneous excitatory and inhibitory post-synaptic current experiments, neurons were held at −70 mV for 10 min to ensure stable recordings, followed by 10 min periods of ethanol treatment and ethanol washout. Additional validation experiments were conducted to confirm that the currents under investigation were mediated by the receptors of interest. sEPSCs were reduced in frequency by ≈93% by 1 mM kynurenic acid, and sIPSCs were reduced in frequency by ≈95% by 50 μM picrotoxin (data not shown).

### Synaptic Plasticity

For LTD synaptic plasticity experiments, recording electrodes were filled with (in mM): 120 K-gluconate, 10 KCl, 10 HEPES, 2 MgCl_2_, 1 EGTA, 2 Mg-ATP, and 0.3 Tris-GTP. Neurons were held at −70 mV for the entirety of the experiment, and the ACSF was supplemented with picrotoxin (50 μM). Standard evoked EPSCs were established for at least 10 min (at 0.025 Hz) to ensure stable recordings, and then followed by a low-frequency stimulation protocol consisting of 1 Hz stimulation for 15 min. Evoked EPSCs were then monitored for a 30 min post-stimulation period at 0.025 Hz to test for the expression of LTD.

### Data Acquisition and Analysis

**Figure 1:**
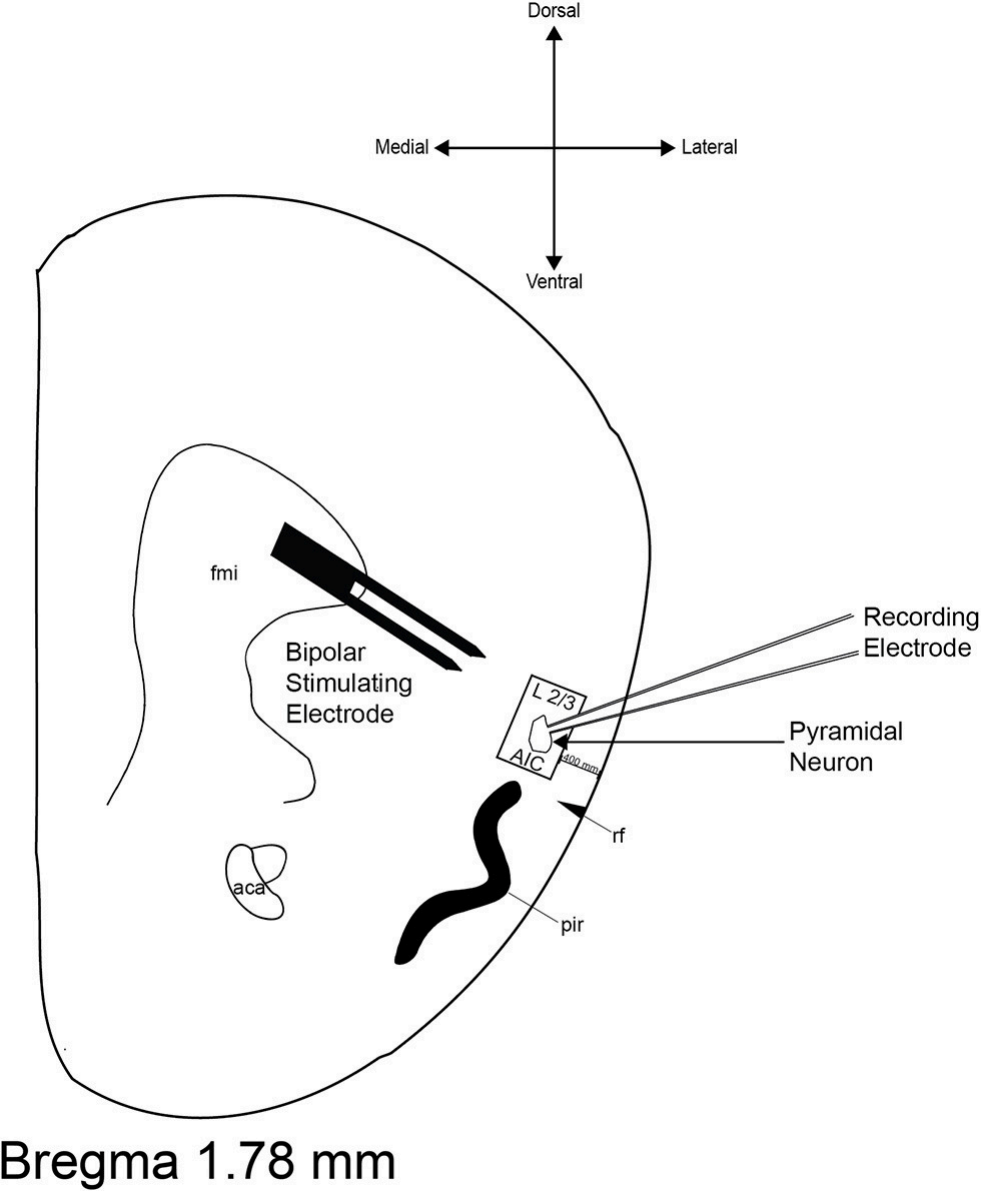
Representative diagram of recording site and bipolar stimulating electrode placement. The boundaries of the area from which neurons were selected for recordings are inside the black box. The boundaries of the stimulating electrode are located ≈500 μm dorsomedial to the recording electrode. aca, anterior commissure; AIC, agranular insular cortex; fmi, forceps minor of the corpus callosum; pir, piriform cortex; rf, rhinal fissure.

All currents were acquired using an Axopatch 200B amplifier (Axon Instruments, Foster City, CA), filtered at 1 kHz, and digitized at 10–20 kHz via a Digidata 1440A interface board using pClamp 10.2 (Axon Instruments). In spontaneous experiments, sEPSCs and sIPSCs were recorded for 30 min and separated into 198 consecutive sweeps; events >5 pA and 10 pA were analyzed in sEPSC and sIPSC experiments, respectively, for mean frequency and mean amplitude. For all evoked experiments, post-synaptic currents (100–200 pA) were evoked via either theta glass electrode or a stainless steel bipolar stimulating electrode (MX21AES, FHC, Inc., Bowdoin, ME, United States) placed ~500 μm dorsomedial to the cell body (Figure 1).

For all experiments investigating acute ethanol on GABAergic and glutamatergic transmission, we used two approaches to statistical analysis. First, a General Linear Model Repeated Measures in IBM SPSS Advanced Statistics 23 was used, with time or phase of the experiment as the repeated measure, and treatment condition (ethanol concentration) as the between-groups factor. For evoked currents, we analyzed the entire time course of the experiment, with 28 levels of the repeated measure (time), and 4 levels (eNMDAR) or 5 levels (eAMPAR) of the between-groups factor (treatment condition). For spontaneous currents, we used phase of the experiment as the repeated measure (3 levels: baseline, treatment, and washout) and treatment condition as the between-groups factor (2 levels). When sphericity within groups was violated (as indicated by Mauchly's test), the Greenhouse-Geisser adjusted degrees of freedom and *p*-values were reported in the text, rounded to the nearest whole number. Second, we also analyzed the effects of ethanol on evoked and spontaneous currents without the use of a repeated measure. We used a 1-way between groups ANOVA to compare treatment conditions during a particular phase of the experiment—either the treatment phase (for evoked NMDAR) or the washout phase (for evoked AMPAR). These were followed by Bonferroni-corrected multiple comparisons. For spontaneous currents, we performed between groups analysis (*t*-test) to compare treatment conditions during just the treatment phase.

GraphPad Prism 8.0 was used to analyze LTD experiments. The expression of LTD was determined by comparing the 20 to 30 min period after the low-frequency stimulation protocol to the 10 min baseline period. Statistical significance from baseline for within each treatment group was defined as *p* < 0.05 using a one-sample *t*-test. Group comparisons for LTD experiments were made using a one-way ANOVA and Bonferroni *post-hoc* test. Statistical significance for all experiments was defined as *p* < 0.05.

### Mice

Ethanol-naïve *Drd1a*-tdTomato BAC transgenic male mice (MMRRC: 030512-UNC) of at least 7 weeks of age were used for all experiments. Briefly, an existing colony of *Drd1a*-tdTomato mice (Ade et al., 2011; initial breeding pairs obtained from The Jackson Laboratory, Stock No. 016204) was maintained by backcrossing mice onto a C57BL/6J background in which only one parent carried the *Drd1a*-tdTomato transgene (as described in Mangieri et al., 2017). Mice were group-housed (up to five mice per cage) in standard cages (7.5″ × 11.5″ × 5″) with Sani-Chips wood bedding (PJ Murphy) at 22°C with a 12:12 light: dark cycle (lights off at 9:30AM). Water and standard chow (LabDiet®5LL2 Prolab RMH1800) were available *ad libitum*, and all experimental procedures were approved by the University of Texas Institutional Animal Care and Use Committee.

## Results

### Ethanol-Sensitivity of Evoked NMDA-Type Glutamatergic Transmission

**Figure 2:**
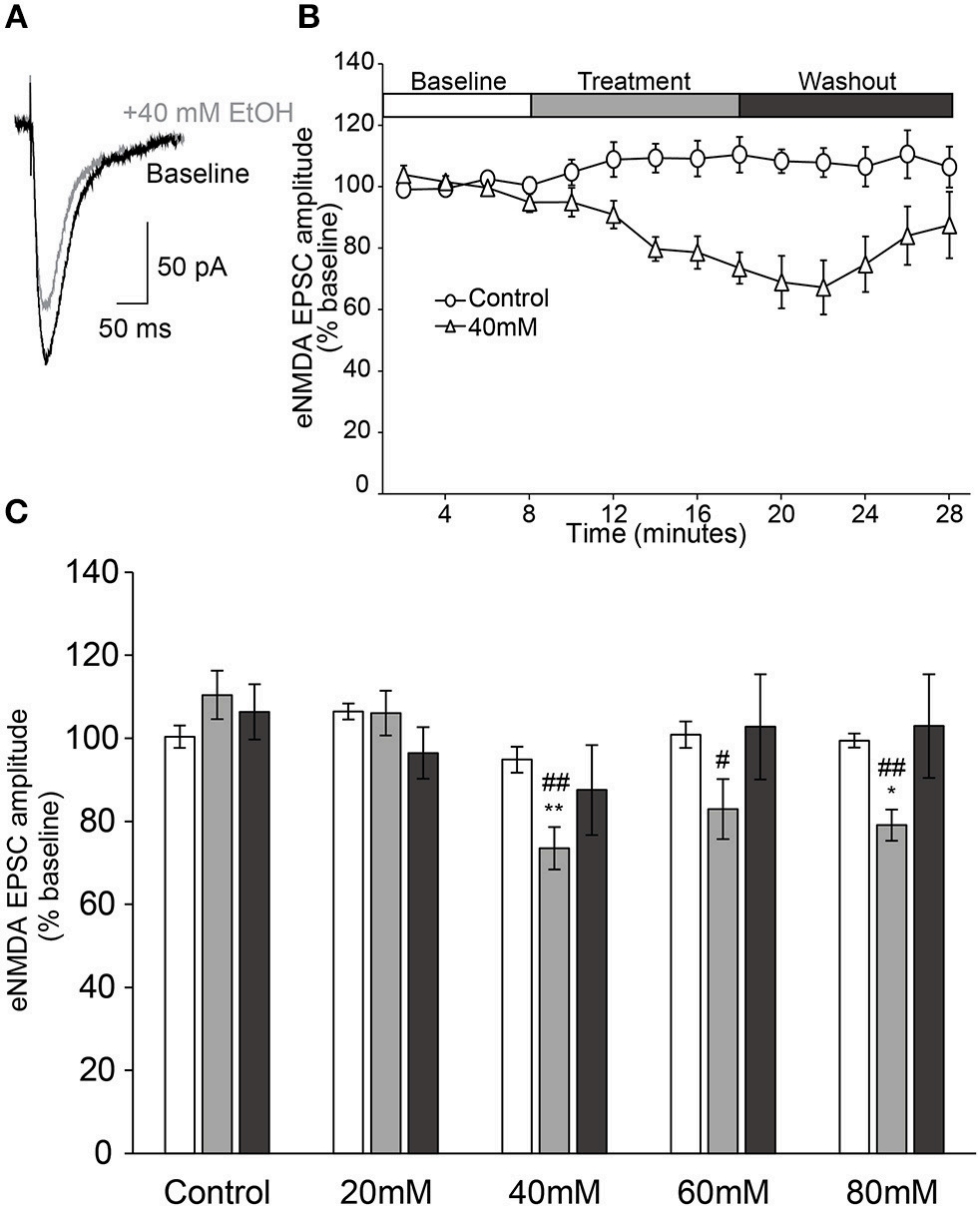
Ethanol inhibits evoked NMDAR-mediated EPSCs in AIC layer 2/3 pyramidal neurons. **(A)** Representative traces from a single neuron showing evoked NMDAR-mediated EPSCs before and after treatment of acute ethanol (40 mM). **(B)** Normalized timecourse of evoked NMDAR EPSC (eNMDA) responses in either sham solution exchange (open circles) or 40 mM ethanol application (open triangles) conditions. The white bar displays the 8 min baseline period, the gray bar displays the 10 min treatment period, and the dark gray bar shows the 10 min washout period. **(C)** Bars show average eNMDA EPSC amplitudes during the last 2 min of each period of the experiment (Baseline, Treatment, Washout), expressed as a percentage of the entire 8 min baseline average. Values are expressed as averages ± S.E.M. ^*^p < 0.05 compared to control during treatment. ^**^p < 0.01 compared to control during treatment. ^#^*p* < 0.05 compared to 20 mM during treatment. ^##^*p* < 0.01 compared to 20 mM during treatment (Control, *n* = 4 neurons/4 slices/2 mice; 20 mM, *n* = 8 neurons/8 slices/4 mice; 40 mM, *n* = 7 neurons/7 slices/4 mice; 60 mM, *n* = 7 neurons/7 slices/4 mice; 80 mM, *n* = 8 neurons/8 slices/4 mice).

NMDAR-mediated currents were evoked for 8 min to ensure steady baseline responses before slices were perfused with an ethanol-containing ACSF for a 10 min treatment period, followed by a 10 min ethanol washout period (Figure 2). When analyzed over the entire 28 min experiment, we observed that the effect of time on NMDAR-mediated EPSC amplitude was not uniform, but varied with the treatment condition [two-way repeated measures ANOVA, main effect of time: *F*_(3, 86)_ = 4.7, *p* = 0.004; time × treatment interaction: *F*_(12, 86)_ = 2.3, *p* = 0.013], indicating that the changes in EPSC amplitude over the course of the experiment were not due to time alone. This conclusion was further supported by a significant between-groups effect during the treatment period [*F*_(4, 29)_ = 8.1, *p* < 0.001], when higher concentrations of ethanol (≥40 mM) all displayed significant reduction of peak NMDAR-mediated response relative to control (Figure 2C).

### Ethanol-Sensitivity of Evoked AMPAR-Type Glutamatergic Transmission

Ethanol has been shown to inhibit NMDAR-mediated currents across several brain regions, but has also been shown to inhibit AMPAR-mediated currents (Lovinger and Roberto, 2013). In order to test the sensitivity of AMPAR-mediated currents to ethanol, we tested whether ethanol modulated evoked AMPAR-mediated transmission. Therefore, neurons were voltage-clamped at −70 mV, and EPSCs were evoked in the presence of 100 μM DL-APV and 50 μM picrotoxin to isolate AMPAR-mediated currents. Following 8 min of recording to ensure steady baseline responses, neurons were perfused with an ethanol-containing ACSF for a 10 min treatment period, followed by a 10 min ethanol washout period.

**Figure 3:**
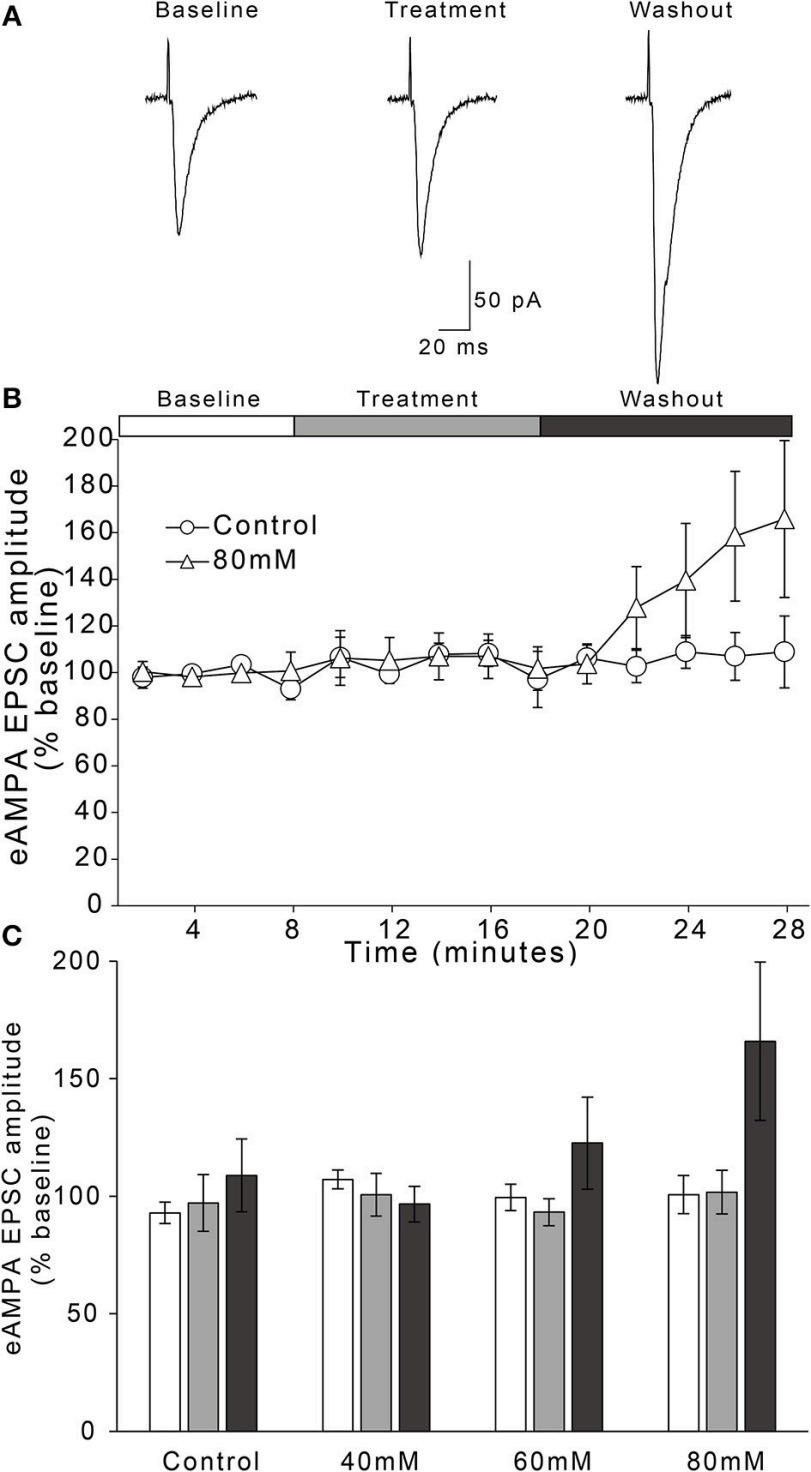
Ethanol has no effect on evoked AMPAR-mediated EPSC amplitudes in AIC layer 2/3 pyramidal neurons. **(A)** Representative traces from a single neuron showing evoked AMPAR-mediated EPSCs before and after treatment with acute ethanol (80 mM). **(B)** Normalized timecourse of evoked AMPAR EPSC (eAMPA) responses in either sham solution exchange (open circles) or 80 mM ethanol application (open triangles) conditions. The white bar displays the 8 min baseline period, the gray bar displays the 10 min treatment period, and the dark gray bar shows the 10 min washout period. **(C)** Bars show average eAMPA amplitudes during the last 2 min of each period of the experiment (Baseline, Treatment, and Washout), expressed as a percentage of the entire 8 min baseline average. Values are expressed as averages ± S.E.M (Control, *n* = 4 neurons/4 slices/3 mice; 40 mM, *n* = 6 neurons/6 slices/4 mice; 60 mM, *n* = 5 neurons/5 slices/4 mice; 80 mM, *n* = 9 neurons/9 slices/7 mice).

We observed no effect of time or time × treatment condition interaction on AMPAR-mediated EPSC amplitude over the entire 28 min experiment [Figure 3; two-way repeated measures ANOVA, main effect of time: *F*_(2, 37)_ = 1.9, n/s; time × treatment interaction: *F*_(6, 37)_ = 1.7, n/s]. In the 60 and 80 mM treatment concentrations, there appeared to be a delayed enhancement of peak AMPAR-mediated EPSCs during the last 2 min of the 10 min washout period (Figure 3C). However, one-way ANOVA comparing treatment conditions during this time period indicated these enhancements were not statistically significant [*F*_(3, 20)_ = 1.44, n/s].

### Ethanol-Sensitivity of sEPSCs

As a final assay of whether ethanol modulates glutamatergic transmission onto layer 2/3 AIC pyramidal neurons, we tested whether acute ethanol modulates spontaneous EPSCs (sEPSCs) in the AIC. Ethanol has been shown to reduce presynaptic glutamate release in multiple brain regions (Lovinger and Roberto, 2013). We assumed that any ethanol-induced changes in sEPSC frequency would be indicative of changes in presynaptic glutamate release, while ethanol-induced changes in sEPSC mean amplitude would be indicative of changes in post-synaptic sensitivity to glutamate (Siggins et al., 2005). Therefore, neurons were voltage-clamped at −70 mV, and sEPSCs were recorded in the presence of 50 μM picrotoxin to yield glutamate receptor-mediated spontaneous currents. Following 10 min of recording to ensure steady baseline responses, neurons were perfused with an ethanol-containing ACSF for a 10 min treatment period, followed by a 10 min ethanol washout period.

**Figure 4:**
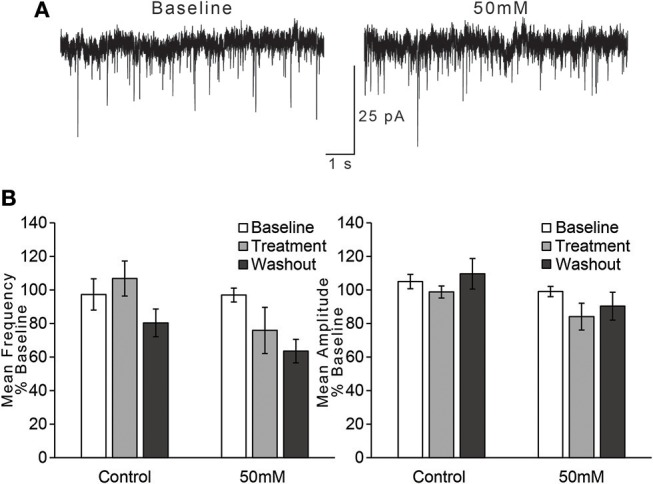
Ethanol has no effect on spontaneous EPSCs in AIC layer 2/3 pyramidal neurons. **(A)** Representative traces from a single neuron showing spontaneous EPSCs (sEPSCs) before and after treatment with acute ethanol (50 mM). **(B)** Summary charts showing mean frequency (left) and amplitude (right) of sEPSCs during the last 2 min of each period of the experiment (Baseline, Treatment, and Washout), expressed as a percentage of the entire 10 min baseline average. Values are expressed as averages ± S.E.M (Control, *n* = 8 neurons/8 slices/6 mice; 50 mM, *n* = 9 neurons/9 slices/7 mice).

For the effect of ethanol on mean frequency of sEPSCs, we observed that an effect of time on sEPSC frequency did not vary by treatment condition [Figure 4; two-way repeated measures ANOVA, main effect of time: *F*_(2, 30)_ = 4.2, *p* = 0.025; time × treatment interaction: *F*_(2, 30)_ = 1.41, n/s]. We also directly compared the two treatment conditions (control vs. 50 mM ethanol) during just the treatment phase of the experiment, but this analysis also did not indicate a statistically significant effect of ethanol: *t*_(15)_ = 1.756, n/s. Thus, although sEPSC frequency appeared to decrease with the application of 50 mM ethanol, the magnitude of this change was not different than that observed in the control treatment group. For the effect of ethanol on mean amplitude of sEPSCs, we observed no effect of time or interaction of time with treatment condition [Figure 4; two-way repeated measures ANOVA, main effect of time: *F*_(2, 30)_ = 2.3, n/s; time × treatment interaction: *F*_(2, 30)_ = 0.84, n/s], nor a difference in treatment conditions during the treatment period: *t*_(15)_ = 1.607, n/s.

### Ethanol-Sensitivity of sIPSCs

In addition to our investigation of the effects of acute ethanol on glutamatergic transmission, we wished to characterize whether ethanol modulates GABAergic transmission in the AIC. Acute ethanol has been shown to modulate GABAergic transmission in several brain regions and experimental preparations (Lovinger and Roberto, 2013). Therefore, we tested whether acute ethanol modulated GABA_A_R-mediated spontaneous IPSCs (sIPSCs) in the AIC. Neurons were voltage-clamped at −70 mV, and sIPSCs were recorded in the presence of 1 mM kynurenic acid to block glutamatergic transmission. Following 10 min of recording to ensure steady baseline responses, neurons were perfused with an ethanol-containing ACSF for a 10 min treatment period, followed by a 10 min ethanol washout period.

**Figure 5:**
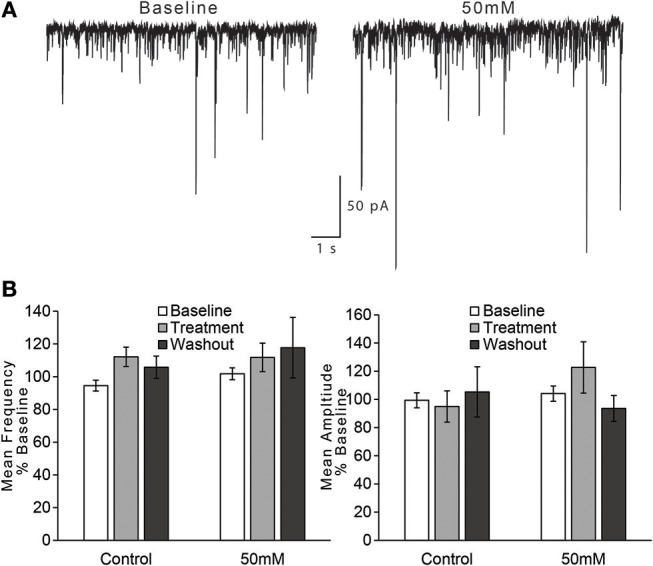
Ethanol has no effect on spontaneous GABA IPSCs in AIC layer 2/3 pyramidal neurons. **(A)** Representative traces from a single neuron showing spontaneous IPSCs (sIPSCs) before and after treatment with acute ethanol (50 mM). **(B)** Summary charts showing mean frequency (left) and amplitude (right) of sIPSCs during the last 2 min of each period of the experiment (Baseline, Treatment, and Washout), expressed as a percentage of the entire 10 min baseline average. Values are expressed as averages ± S.E.M (Control, *n* = 6 neurons/6 slices/6 mice; 50 mM, *n* = 11 neurons/11 slices/9 mice).

For the effect of ethanol on mean frequency of sIPSCs, we observed no effect of time on mean frequency of sIPSCs and no time × treatment condition interaction [Figure 5; two-way repeated measures ANOVA, main effect of time: *F*_(1, 18)_ = 1.52, n/s; time × treatment interaction: *F*_(1, 18)_ = 0.24, n/s]. We also found no difference between treatment conditions (control vs. 50 mM ethanol) during the treatment phase: *t*_(15)_ = 0.031, n/s. For the effect of ethanol on mean amplitude of sIPSCs, we observed no effect of time on mean amplitude of sIPSCs and no time × treatment condition interaction [Figure 5; two-way repeated measures ANOVA, main effect of time: *F*_(2, 30)_ = 0.43, n/s; time × treatment interaction: *F*_(2, 30)_ = 1.79, n/s]. A separate comparison of just the treatment phase of the experiment also did not reveal a statistically significant difference between control and 50 mM ethanol: *t*_(15)_ = 1.058, n/s.

### LTD in AIC Layer 2/3 in Ethanol-Naïve Mice

**Figure 6:**
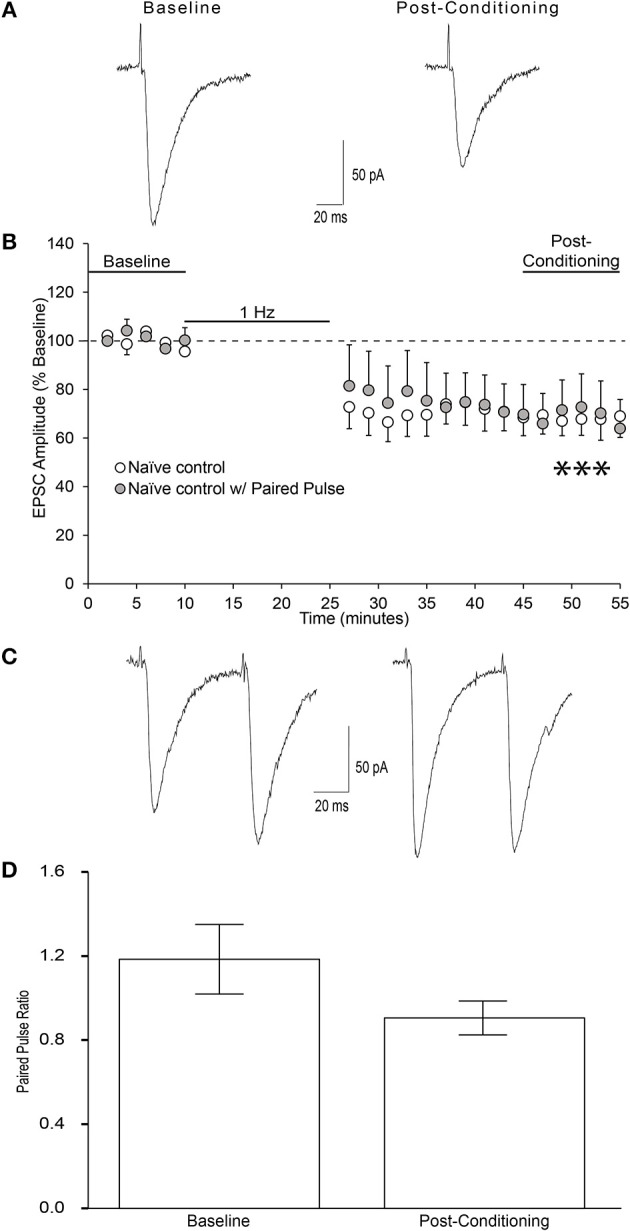
Low-frequency stimulation produces LTD at glutamatergic synapses onto AIC layer 2/3 pyramidal neurons. **(A)** Representative traces from a single neuron showing evoked EPSCs before and 20–30 min after low-frequency conditioning stimulation protocol (900 pulses at 1 Hz while holding the neuron at −70 mv). **(B)** Conditioning stimulation induced long-term depression of evoked EPSCs onto AIC layer 2/3 pyramidal neurons of ethanol-naïve mice (12 neurons/12 slices/9 mice, ^***^p = 0.0007 compared to baseline). **(C)** Representative traces from a single neuron showing evoked paired pulse ratios before baseline and after the post-conditioning period. **(D)** Bar graph representing the mean PPR ± S.E.M. before baseline and after the post-conditioning period. PPR was determined by dividing the amplitude of EPSC2 by EPSC1 for each sweep. Average PPRs before baseline and after post-conditioning were not significantly different (*n* = 6 neurons/6 slices/4 mice; paired *t*-test, *t* = 2.48, n/s). Values are expressed as averages ± S.E.M.

We found that in the presence of 50 μM picrotoxin, local low frequency stimulation (1 Hz for 15 min) induced long-term depression (LTD) of evoked EPSCs in layer 2/3 AIC pyramidal neurons (Figures 6A,B; one-sample *t*-test, *t* = 4.622, *p* = 0.0007). To investigate whether the reduction in EPSC magnitude observed was due to either presynaptic changes in glutamatergic release or post-synaptic changes in glutamate receptor sensitivity, we measured paired-pulse ratios (2 pulses, 50 ms apart) before the 10 min of baseline recording and after the 30 min of post-stimulation in a separate group of neurons. Neurons tested for paired-pulse ratios displayed equivalent post-conditioning EPSC amplitudes to that seen in naïve control neurons (Figure 6B; unpaired *t*-test, *t* = 0.05, n/s). We observed no change in paired-pulse ratios before and after the induction of LTD, indicating that LTD in layer 2/3 AIC neurons is not due to changes in presynaptic glutamate release (Figures 6C,D; paired *t*-test, *t* = 2.484, n/s).

### LTD in AIC Layer 2/3 in Ethanol-Naïve Mice Is NMDAR-Dependent and Ethanol Sensitive

**Figure 7:**
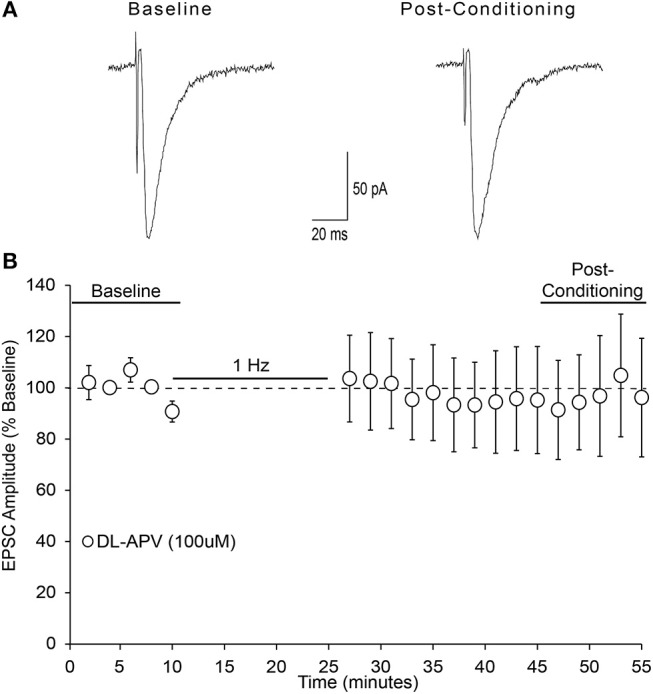
NMDA receptors are required for AIC layer 2/3 pyramidal neuron LTD expression. **(A)** Representative traces from a single neuron showing evoked EPSCs before and 20–30 min after low-frequency conditioning stimulation protocol in the presence of the non-selective NMDA receptor antagonist DL-APV (100 μM). **(B)** Conditioning stimulation did not induce LTD expression in the presence of DL-APV (100 μM), (7 neurons/7 slices/6 mice, *p* > 0.05 vs. baseline). Values are expressed as averages ± S.E.M.

An investigation from Liu and colleagues was the initial demonstration and investigation of LTD in the mouse insular cortex (IC) (Liu et al., 2013). They found via field potential recordings that low frequency stimulation in adult mouse IC can induce LTD of evoked excitatory post-synaptic potentials that depends upon NMDAR activation. Since this is the first investigation of whole cell LTD in the IC, we wished to determine whether our observed form of LTD similarly depended upon NMDAR activation. Bath application of the non-selective NMDA receptor antagonist DL-APV (100 μM) blocked the expression of LTD (Figure 7; one-sample *t*-test, *t* = 0.154, n/s).

**Figure 8:**
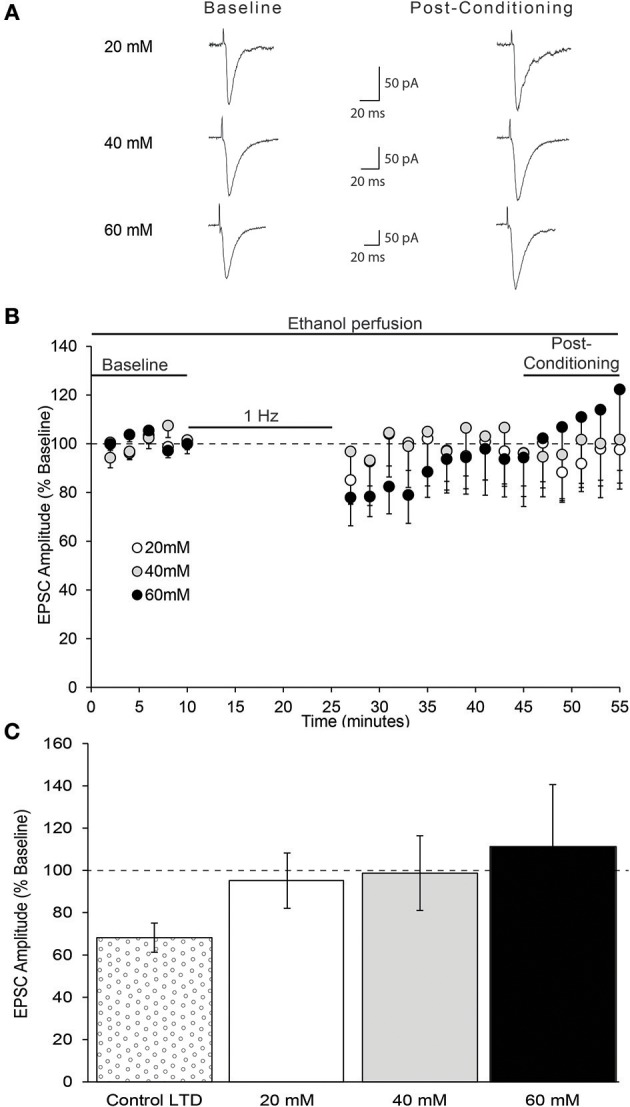
*In vitro* ethanol exposure blocks AIC layer 2/3 pyramidal neuron LTD expression. **(A)** Representative traces from a single neuron of each ethanol group showing evoked EPSCs before and 20–30 min after low-frequency conditioning stimulation protocol in the presence of acute ethanol. **(B)** Conditioning stimulation did not induce LTD expression in the presence of acute ethanol (20, 40, or 60 mM). **(C)** Bar graph representing the average post-conditioning (min 45–55) EPSC amplitude as percentage of baseline for each ethanol concentration. Control LTD value from prior experiment (Figure 6) shown for comparison. Values are expressed as averages ± S.E.M (Control LTD, *n* = 12 neurons/12 slices/9 mice; 20 mM, *n* = 7 neurons/7 slices/5 mice; 40 mM, *n* = 6 neurons/6 slices/5 mice; 60 mM, *n* = 7 neurons/7 slices/5 mice).

Acute ethanol has been shown to modulate the expression NMDAR-dependent forms of synaptic plasticity in multiple brain regions (McCool, 2011). Therefore, we next tested whether acute pharmacologically relevant concentrations of ethanol modulate the expression of layer 2/3 AIC pyramidal neuron LTD. Bath application of multiple concentrations of ethanol (20, 40, 60 mM) did not differ in their ability to block the expression of AIC LTD [Figure 8; one-way ANOVA, *F*_(2, 17)_ = 0.16, n/s].

## Discussion

### Ethanol Has Multiple Effects on the Glutamate System

The major findings of this investigation are that acute ethanol has significant effects on glutamatergic transmission and glutamatergic synaptic plasticity in layer 2/3 AIC pyramidal neurons, but little to no effect on GABAergic transmission at the concentration tested (50 mM). Recordings from brain slice preparations across multiple brain regions have generally shown an inhibitory effect of acute ethanol on glutamatergic transmission (Lovinger and Roberto, 2013). This effect is largely attributed to ethanol's inhibitory actions on post-synaptic NMDARs (Ron and Wang, 2009). Since acute ethanol has been shown to modulate glutamatergic transmission across several brain regions and experimental preparations (Lovinger and Roberto, 2013), we wished to determine whether ethanol modulates glutamatergic intracortical processing in the AIC. A commonly replicated synaptic effect of ethanol across multiple brain regions has been its inhibitory action on post-synaptic NMDARs, as ethanol has generally been found to have a concentration-dependent inhibition of NMDAR-mediated transmission (Ron and Wang, 2009). Moreover, ethanol's inhibitory effects on NMDARs and disruption of NMDAR-dependent signaling processes have been shown to be major canonical mechanisms by which chronic ethanol disrupts healthy brain functioning; NMDAR-dependent synaptic mechanisms of learning and memory have generally been shown to be disrupted by chronic alcohol use and implicated in alcohol-related phenotypes (Ron and Wang, 2009). For these reasons we tested whether ethanol inhibited post-synaptic NMDARs in layer 2/3 AIC pyramidal neurons. Our investigation determined that ethanol modestly inhibited evoked NMDAR-mediated currents in the AIC in a concentration-related manner. Such a finding complements research in other brain regions which identifies NMDARs as a modest (≈ 25% inhibition) ethanol-sensitive target in cortical neurons (Lovinger and Roberto, 2013). However, it is important to consider that no statistically significant inhibition of evoked NMDAR-mediated EPSCs was observed at 20 mM, an intoxicating ethanol concentration. Therefore, our data, at initial consideration, suggest that the action of ethanol on NMDARs in the AIC is a modest effect observable only at highly intoxicating concentrations (≥40 mM) of ethanol.

However, it is possible that ethanol's action on AIC NMDARs *in vivo* occurs at lower ethanol concentrations and at greater peak inhibition levels than what we observed in the current study due the limitations of a brain slice preparation. For example, a well-established modulator of the degree of ethanol inhibition on evoked NMDAR-mediated responses is the ACSF Mg^2+^ concentration, as studies have shown that higher Mg^2+^ concentrations increase the sensitivity of NMDARs to ethanol (Carlton et al., 1998; Ron and Wang, 2009). Prior research investigating ethanol's inhibitory effect on NMDARs utilizing expression systems has shown that the degree of ethanol inhibition of NMDARs is Mg^2+^-dependent (Jin et al., 2008). Our experimental design utilized a concentration of Mg^2+^ (1.0 mM) that has been shown to produce significant inhibition of evoked NMDAR currents of pyramidal neurons in the basolateral amygdala (Carlton et al., 1998). Since normal cerebrospinal Mg^2+^ concentration in healthy people is estimated to be around 1.48 mM, due to enhanced free Mg^2+^ in human cerebrospinal fluid, AIC NMDARs may be more sensitive to the inhibitory effects of ethanol under physiological conditions than under those of our brain slice preparation (Banki et al., 1985).

Nonetheless, even if the modest level of inhibition observed only at higher ethanol concentration levels (40, 60, 80 mM) in this study fully replicate *in vivo* conditions, we still maintain that this inhibitory effect is a significant phenomenon by which chronic ethanol exposure likely elicits long-term alterations AIC functioning. NMDARs that display sensitivity to acute ethanol inhibition generally enhance their functioning in response to chronic ethanol exposure as a compensatory mechanism due to ethanol's chronic inhibition, which results in aberrations from homeostatic NMDAR-dependent signaling processes (Roberto and Varodayan, 2017). Prior research from our laboratory, among others, has shown that these long term alterations due chronic ethanol exposure lead to robust changes in expression of NMDAR-dependent plasticity states and ethanol-related behavior (Jeanes et al., 2011, 2014; Abrahao et al., 2013). Thus, our findings suggest that NMDARs and NMDAR-mediated signaling processes in layer 2/3 AIC pyramidal neurons are ethanol-sensitive targets likely to underlie alterations in AIC function after chronic ethanol exposure. Since layer 2/3 is the intracortical processing layer of the AIC, our data suggest that general intracortical processing in the AIC as well as its output to downstream brain regions are sensitive to disruption by chronic ethanol.

In order to test the sensitivity of AMPAR-mediated glutamatergic transmission to ethanol, we examined whether evoked AMPAR-mediated currents were sensitive to ethanol. Our investigation found that evoked AMPAR-currents were insensitive to intoxicating concentrations of ethanol, except for a non-statistically significant delayed enhancement nearly 20 min after the initial bath application of ethanol at a concentration nearly lethal (80 mM) to intolerant individuals. As such, these negative results on evoked AMPAR-mediated currents are indicative of a selective post-synaptic action of ethanol. However, as a final test of ethanol action on presynaptic glutamate release, we measured whether acute ethanol modulated sEPSCs. We found that the significantly intoxicating concentration of ethanol (50 mM) did not change the mean frequency or mean amplitude of sEPSCs, indicative of no changes in glutamate release probability.

In summary, these findings contribute to the abundance of literature indicating that the effects of acute ethanol on glutamatergic transmission in brain slice preparations are brain-region specific and concentration dependent. Acute ethanol has been shown to generally reduce glutamatergic transmission (Lovinger and Roberto, 2013). However, investigations of acute ethanol on glutamatergic transmission in some brain regions, such as the ventral tegmental area, somatosensory cortex, and central amygdala show an ethanol-induced enhancement of glutamatergic transmission (Lu and Yeh, 1999; Xiao et al., 2009; Silberman et al., 2015; Herman et al., 2016).

### Ethanol Has Little Action on GABA_A_ Transmission

Similar to the glutamate system, modulatory effects of ethanol on GABA_A_-mediated transmission in brain slice preparations have depended upon the brain region investigated as well as the ethanol concentration used (Nie et al., 1994; Lu and Yeh, 1999; Roberto et al., 2004). Acute ethanol has generally, but not always been shown to increase GABAergic transmission by both pre and post-synaptic mechanisms (Siggins et al., 2005; Lovinger and Roberto, 2013). However, some studies have shown that GABAergic transmission in cortical regions is relatively insensitive to acute ethanol (Proctor et al., 1992; Soldo et al., 1998; Weitlauf and Woodward, 2008). The current investigation did not show any effects of ethanol on spontaneous GABA_A_-mediated transmission. We therefore conclude from our investigation that an intoxicating concentration of ethanol has little, if any, effect on spontaneous GABA_A_-mediated transmission onto layer 2/3 AIC pyramidal neurons.

### Ethanol Disrupts NMDAR-Dependent Synaptic Plasticity

Since the disrupted processing of interoceptive stimuli has been suggested to play a role in drug and alcohol use disorders, and synaptic plasticity mechanisms are accepted as underlying aspects of learning and memory, we wished to investigate the effect of ethanol on long-term synaptic plasticity in intracortical processing layers of the AIC. We initially found that acute ethanol inhibits NMDARs in the AIC, and so we hypothesized that any NMDAR-dependent long-term synaptic plasticity measures onto layer 2/3 AIC pyramidal neurons would likely be disrupted by acute ethanol. Therefore, we investigated LTD as a long term synaptic plasticity mechanism onto layer 2/3 AIC pyramidal neurons.

Synaptic plasticity mechanisms are the means by which neural networks adapt to strengthen and weaken their connections to form the basis of information storage and are thought of as mechanisms of learning and memory (Kauer and Malenka, 2007; Kandel et al., 2014). Such synaptic plasticity mechanisms in mesolimbic, addiction-relevant brain regions have been shown to be disrupted by drug experience and are thought to encode for and contribute to future drug and alcohol use (Lüscher and Malenka, 2011; Lovinger and Kash, 2015). Since the AIC and its output have been shown, in animal models, to be involved in more advanced, pathological forms of alcohol drinking, we reasoned that ethanol-induced changes in AIC processing and its output may mediate the changes in interoceptive functioning that are implicated in AUD. Therefore, we decided to investigate plasticity mechanisms in AIC layer 2/3 pyramidal neurons. We performed the first demonstration of LTD using whole cell configuration in the IC. Using a 1 Hz, low-frequency stimulation protocol, we found a reduction in EPSC magnitude (LTD) of ~34%. This form of LTD was NMDAR-dependent and likely mediated by a post-synaptic mechanism. Since prior investigation in this study had determined an inhibitory effect of ethanol on NMDARs in AIC 2/3 pyramidal neurons, we reasoned that ethanol may, through its actions on NMDARs, inhibit the expression of our discovered NMDAR-dependent LTD mechanism. We found that AIC LTD was similarly inhibited by several intoxicating concentrations of acute ethanol (20, 40, 60 mM), indicating that this NMDAR-dependent plasticity state is highly sensitive to intoxicating concentrations of ethanol.

It is noteworthy that while 20 mM ethanol prevented the expression of LTD, this concentration of ethanol did not inhibit evoked NMDAR-mediated currents. We suggest there are at least three reasons why this could be so. First of all, the difference in ACSF Mg^2+^ concentration between LTD experiments (1.2 mM) and evoked NMDAR-mediated current experiments (1.0 mM) suggests that NMDARs were sensitive to lower concentrations of ethanol in LTD experiments than in evoked NMDAR-mediated experiments, as higher Mg^2+^ concentrations increase the sensitivity of NMDARs to ethanol (Ron and Wang, 2009). Secondly, it is possible that ethanol inhibits our uncovered form of synaptic plasticity via an alternative molecular target than NMDARs. Ethanol in acute preparations has a wide array of molecular targets, and has been shown to inhibit the expression of forms of LTD via its inhibitory action on synaptic metabotropic glutamate receptors (mGluRs) (Carta et al., 2006; Belmequenai et al., 2008; Su et al., 2010; Zorumski et al., 2014). Generally, the major post-synaptic forms of LTD have been shown to be either NMDAR or mGluR-dependent, but some require both NMDARs and mGluRs (Collingridge et al., 2010). Therefore, it is possible that our uncovered form of AIC LTD was additionally mGluR-dependent, and that acute ethanol inhibited its expression, at least in part, via its inhibitory actions on mGluRs. Finally, NMDARs have metabotropic actions; thus it is possible that this APV-sensitive LTD is not mediated by ion flux (Dore et al., 2016).

### AIC Synaptic Plasticity, Pain, and Alcohol Use Disorder

Recent research suggests that the neurobiological substrates for pain disorders and addiction overlap, and that adaptations in brain regions involved in chronic pain contribute to alcohol use disorder (Egli et al., 2012). Multiple animal models have implicated NMDAR-depending signaling processes in the IC as targets encoding for chronic pain: The ability to induce IC NMDAR-dependent long-term potentiation and the ability to induce IC NMDAR-dependent LTD in *ex vivo* slice preparations were each shown to be lost in animal models of chronic pain (Qiu et al., 2013; Liu and Zhuo, 2014). This evidence of disrupted IC NMDAR-dependent signaling processes in chronic pain considered alongside ethanol's widely demonstrated disruption of NMDAR-dependent signaling processes suggests that NMDAR-dependent signaling processes in the IC may be shared mechanisms by which both pain and ethanol change IC function. In the present work, we verified that layer 2/3 of the AIC is an additional region in which acute ethanol modulates NMDAR function, and we observed that NMDAR-dependent plasticity in the AIC is sensitive to intoxicating concentrations of ethanol used to develop alcohol dependence in animal models. Thus, together these findings suggest that processing in the AIC is sensitive to acute ethanol disruption, and that synaptic mechanisms thought to mediate pain-related interoceptive changes in the AIC can also be disrupted by acute ethanol. This is the initial investigation of the molecular mechanisms by which alcohol exposure may change healthy AIC functioning in the development of AUD.

## Author Contributions

JS and RMo conceived and designed experiments. JS performed the experiments. JS and RMa analyzed the data and interpreted the results. JS and RMa wrote the paper.

### Conflict of Interest Statement

The authors declare that the research was conducted in the absence of any commercial or financial relationships that could be construed as a potential conflict of interest.
